# Supplementary material for: Altered microRNA Transcriptome in Cultured Human Liver Cells upon Infection with Ebola Virus
Source: Int J Mol Sci. 2021 Apr 6;22(7):3792. doi: 10.3390/ijms22073792 (PMC8038836; doi:10.3390/ijms22073792)
Supplement: Supplementary file 1 [file ijms-22-03792-s001.zip › Supplementary_File/C_ GO_Analysis_Results/16-30nt_go_Mayinga-96h-Huh7_vs_Control-96h-Huh7_down.mature_mirna_targets/BP_GeneRatioDotPlot.pdf]

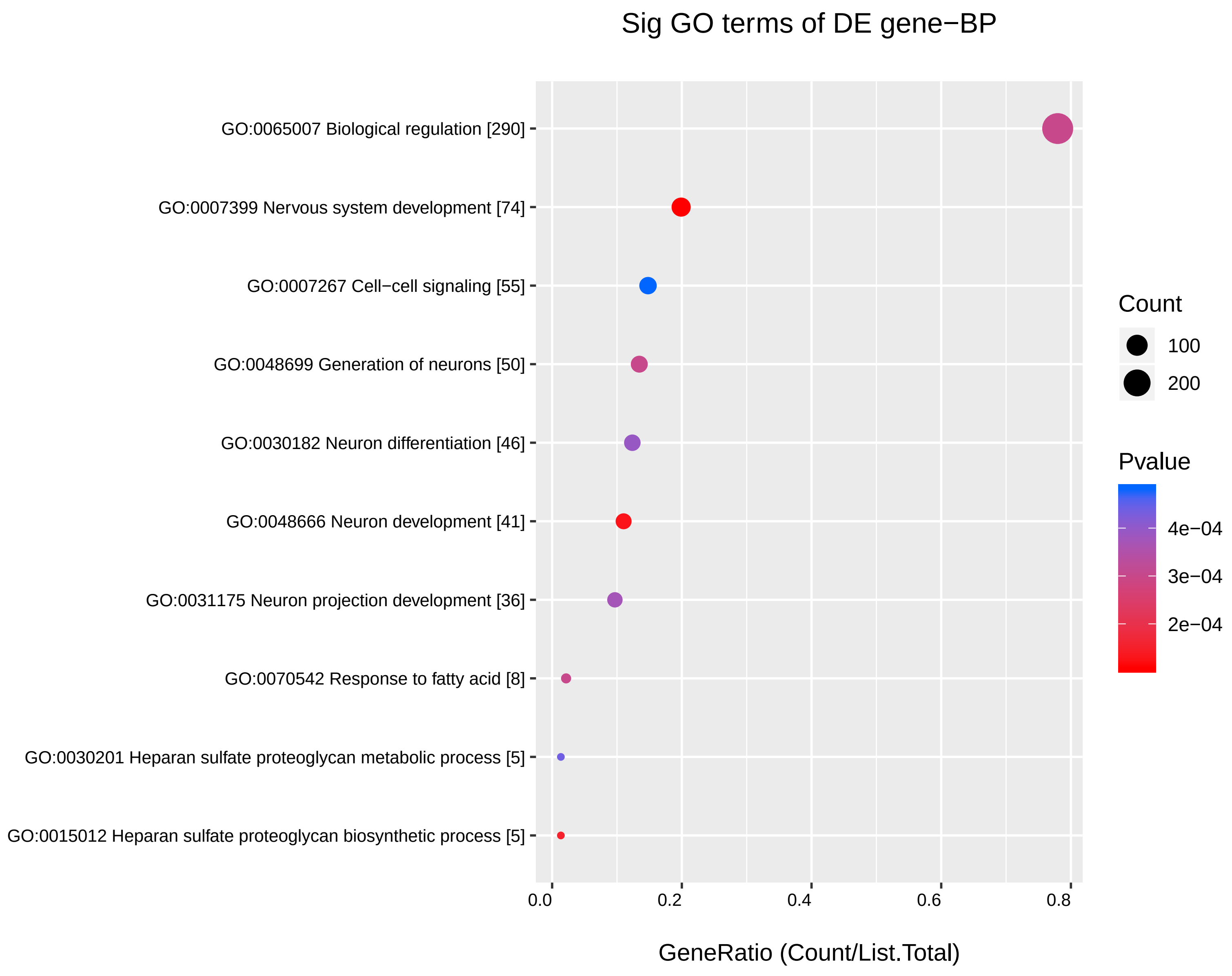

Sig GO terms of DE gene−BP
GO:0065007 Biological regulation [290]
GO:0007399 Nervous system development [74]
GO:0007267 Cell−cell signaling [55]
GO:0048699 Generation of neurons [50]
GO:0030182 Neuron differentiation [46]
GO:0048666 Neuron development [41]
GO:0031175 Neuron projection development [36]
GO:0070542 Response to fatty acid [8]
GO:0030201 Heparan sulfate proteoglycan metabolic process [5]
GO:0015012 Heparan sulfate proteoglycan biosynthetic process [5]
0.0
0.2
0.4
0.6
0.8
GeneRatio (Count/List.Total)
Count
100
200
Pvalue
4e−04
3e−04
2e−04
